# Implicit associations of teleology and essentialism concepts with genetics concepts among secondary school students

**DOI:** 10.1371/journal.pone.0242189

**Published:** 2020-11-20

**Authors:** Florian Stern, Marine Delaval, Kostas Kampourakis, Andreas Müller

**Affiliations:** 1 Faculty of Science, Section of Biology, University of Geneva, Geneva, Switzerland; 2 INSPÉ, Académie de Lille—Hauts de France, Villeneuve d'Ascq, France; 3 PSITEC (EA 4072), Université de Lille, Villeneuve d'Ascq, France; 4 University Teacher Training Institute (IUFE), University of Geneva, Geneva, Switzerland; 5 Faculty of Science, Physics Section, University of Geneva, Geneva, Switzerland; Northeastern University, UNITED STATES

## Abstract

In this article, we present the development and validation of an implicit association test for measuring secondary school students’ associations between genetics concepts and teleology concepts on the one hand, and between genetics concepts and essentialism concepts on the other hand. In total, 169 students from 16 school classes took part in the study, from January 2018 to May 2018. We investigated the strength of the aforementioned associations and the influence of various covariates such as gender, age, school class, or previous learning of biology on the association of teleology or essentialism concepts with genetics concepts through an analysis of covariance and a multi-level analysis. We found moderate associations between genetics and teleology concepts, as well as between genetics and essentialism concepts. These results might reflect a tendency of students of different ages and with various backgrounds to think about genes in terms of goals (teleology) and stability (essentialism), which should be investigated further in future research.

## Introduction

A main goal of science teaching is conceptual understanding. To achieve this, it is not sufficient to simply present science concepts, which are mental representations of the world [1], to students. Rather, teaching should first and foremost address in an explicit and reflective manner students’ conceptions, that is, the different meanings associated with particular concepts [2]. Research in science education has shown that students hold a variety of conceptions related to particular concepts. What is most important is that students’ conceptions often stem from deep intuitions, i.e. spontaneous and subconscious ways of thinking about phenomena, that are strongly held, often persisting into adulthood [3], and in some cases they are not completely overwritten even by expert knowledge [4,5]. These intuitions can make scientific concepts and explanations seem counterintuitive [6]. Of course, not all intuitions hinder learning of scientific concepts, as e.g. some of them lead even young children to grasp sophisticated correlational and causal patterns [7]. However, some intuitions generate persistent conceptions that turn to conceptual obstacles: conceptions that are strongly held, resistant to change, and impede understanding of scientific concepts.

The interplay between intuitive thinking and students’ conceptions and explanations about biological entities and processes has been investigated in cognitive science research [8–12] and in science education [13,14]. Previous research aimed at answering important questions such as whether it is knowledge about artifacts that influences knowledge about organisms or the opposite [15,16]; or whether causes are perceived by humans as central in the process of explanation [17,18]. Biology education research has focused on particular intuitions such as teleology (the idea that the characteristics of organisms exist for a given purpose or goal), psychological essentialism (the idea that organisms have deep underlying essences), or genetic essentialism (the idea that genes are the essences of organisms, specifying what they are). These intuitions are widely used to make sense of phenomena from everyday life. Several studies have provided insights about how teleological [19], essentialist [20], and genetic essentialist intuitions [21] can form obstacles in students’ conceptual understanding in biology. Teleological and essentialist thinking have even been found to be used by instructors at the university level, according to a recent study in 90 university science classes [22].

Several past studies have shown that genetics is difficult to understand because students hold a variety of misconceptions about the nature of the genetic material, about the role of genes and the nature and potential of genetic technologies. These misconceptions, in turn, stem from a variety of sources such as the media, textbooks, teachers themselves and intuitive ways of thinking such as genetic essentialism [23]. It has been well-established in research that people form an intuitive understanding of heredity from a very young age, and therefore develop preconceptions about how the related phenomena occur [24].

Following this body of research about genetics and intuitions, in the present study, we have focused on secondary school students’ conceptions in the context of genetics, and how they relate to teleology and essentialism. There are at least two reasons for this; first, as in many countries formal genetics education starts in secondary school, it is useful to know whether genetic teleology (the intuition that genes exist for a particular purpose or goal) and genetic essentialism conceptions are already present at this level, in order to address them as soon as possible; second, if such conceptions are found, acquiring a more detailed view of them, and of the respective intuitions, can be very useful for designing teaching interventions aiming at conceptual understanding. In the present study, we therefore contribute to the body of research that has documented genetic essentialism misconceptions, such as "the fate of every human being lies in his or her genes" [25], or " […] genes associated with a well-functioning brain always remain fixed" [26]; as well as the genetic teleology intuitions, such as "genes turn on so that a cell can develop properly" [27], or " […] genes associated with a big brain have been designed for several roles such as solving complex problems" [26].

We have therefore developed a test that could be used to investigate whether these intuitions (teleology and essentialism) are related to genetics, and in the present article we report its development in detail. This study is part of a larger project that aims to explore more systematically the links between teleology, essentialism and genetics. Besides assessing students’ *explicit* genetic teleology and genetic essentialism conceptions with a questionnaire we have developed [26], we also wanted to see whether students make *implicit* associations between genetics concepts and particular teleology and essentialism concepts. In the present article we present the development of the implicit association test that we used for this purpose and our findings.

## Research background

### Implicit measures

Implicit measures can reveal attitudinal or cognitive biases or intuitions that people sometimes will not express in an explicit manner, fully or at all [28]. Measures are implicit if participants: 1) are not aware of the fact that the attitude or cognition in question is being measured; 2) do not have conscious access to an attitude or cognition; 3) have no or little control over the measurement outcome [29]. Widely used for several decades in the area of social and personality psychology [28,30], e.g. for implicit attitudes [31,32] and stereotypes [33,34], implicit measures have more recently shed new light on conceptual understanding [35–37]. A widespread experimental paradigm are reaction (or response) time tasks [38–40]. In science education, implicit measures have been used in various ways to reveal intuitions and misconception-like answers that would not likely appear with classic explicit measures [41].

In one strand of research, speeded tasks related to the understanding of different concepts (e.g. “a heavier object sinks faster” [42], or “humans are descended from chimpanzees” [41]) were used to show patterns of answers revealing traces of intuitions that remain intact even after teaching. The results showed that participants consistently answered more rapidly and more accurately for statements in accordance with their intuitive conceptions, across a wide variety of science topics: liquid and solid [43], living beings [44], kinematics graphs [35], and buoyancy [42], or [41] as paradigmatic and cross-sectional study for several topics in the life and physical sciences. The authors in [41] interpreted their findings by suggesting that “scientific knowledge suppresses but does not supplant earlier intuitions”, and that in the case of statements not in accordance with a given intuition the coexistence of the two conflicting conceptions leads to longer processing times and higher errors rates. Other authors have also emphasized that the persistence of intuitive conceptions and their coexistence with the scientific ones is visible in implicit measures, even after teaching on the topic in question and after a good understanding on the explicit level is reached [36]. A later study confirmed this even with science teachers [45]. One of the existing implicit instruments is the Implicit Association Test, which we discuss in the next section.

### The implicit association test

The Implicit Association Test (IAT) has provided another type of speeded response tasks, probing specifically for mental associations. Widely used in social psychology [28,46,47], evidence has been provided to establish it as sufficiently reliable and valid [48–50]. In the context of science education, it has been used to show the prevalence of gender stereotypes related to physics [34].

The IAT measures the differential association of two contrasted target concepts that appear in a 2-choice task (e.g. “flowers” versus “insects”) with two contrasted attributes that appear in a second task (e.g. “good” words versus “bad” words). In the test, words successively appear in the center of the screen, belonging either to one of the target categories (for example, ‘*rose*’ or ‘*bugs*’) or to one of the attribute categories (for example, ‘*happy*’ or ‘*ugly*’). Participants are then asked to classify words using the appropriate keys, as fast as possible, and the latency (the time required for a participant to classify a given word) for each word is recorded. The IAT actually alternates *practice* and *test* trials. The former are designed in order for participants to get used to the key assignment of each category and its left/right switching, whereas the latter are designed in order to measure associations based on the recording of response times. The standard IAT design consists of 5 blocks [51]. In practice trials (blocks 1, 2 and 4), only one contrast (e.g. *‘flowers’* vs *‘insects’*) of categories is displayed. In test trials (blocks 3 and 5), a mix of two attributes and two targets (e.g. *‘good’ + ‘flowers’ vs ‘bad’ + ‘insects’*) is shown.

In order to show whether some associations are preferred over others, latencies from blocks 3 and 5 are compared using a specific indicator for each participant, the D-score. It is defined as D = (IRL-CRL)/SD [52], where:

- CRL (“Compatible response latency”) is the average latency for trials in block 3 that were expected to be associated, such as ‘*flowers*’ and ‘*good*’ words;
- IRL (“Incompatible response latency”) is the average latency for trials in block 5 that were not expected to be associated, such as ‘*flowers*’ and ‘*bad*’ words;
- SD is the standard deviation of latencies calculated across the compatible and incompatible trials.

D-scores greater than 0 could be interpreted as evidence of an association between the compatible categories (e.g. ‘*flowers*’ and ‘*good*’ or ‘*insects*’ and ‘*bad*’). In contrast, D-scores smaller than 0 could be interpreted as evidence of an association between the incompatible categories (e.g. ‘*flowers*’ and ‘*bad*’ or ‘*insects*’ and ‘*good*’) [51,52].

Even though specifically conceived for the study of associations of social behaviors and attitudes, the IAT has however been little used so far to investigate associations related to scientific concepts. To the best of our knowledge Gould and Heine [53] have carried out the first and only study of this kind, providing evidence for implicit associations between gene concepts and fate concepts. We agree with them that IAT studies can help “to develop a rich understanding” [53] of associative networks behind complex scientific concepts and theories such as genetics. In our study, we developed an IAT that measures the differential association of two target concepts (concepts related to genetics vs. concepts related to environment) with an attribute that appears in a second task (words indicating goal vs. words indicating chance in the case of teleology, and words indicating stability vs. words indicating change in the case of essentialism). The IAT is designed to test particular hypotheses; for instance, if the hypothesis were that genetics concepts and teleology concepts were associated, participants would be expected to perform faster when instructions pointed to categories that seem to be highly associated (e.g. “genetics” + “goal”) than when they pointed to two less associated categories (e.g., genetics + chance). Such a difference in performance measures a differential, implicit, association of the two concepts with the attribute. We now turn to the constructs that were of interest to us: genetic teleology and genetic essentialism.

### Genetic teleology

Teleology is the idea that characteristics or properties exist for a purpose; and design teleology is the intuition that this contribution is the outcome of intention or design [54]. In daily life, teleological language is very common as it helps us make sense of the world. It is often based on goal-related words, such as “*for*”, or “*in order to”* (e.g., “a knife is designed *in order to* cut” or “*for* cutting”), and research has shown that students may simply prefer it because they find it familiar and intuitive [55]. Teleological thinking is perfectly legitimate for artifacts. For example, we can confidently state that “airplanes have wings in order to fly”; artifacts have specific features for intended uses because they were intentionally designed for this purpose. However, this notion does not always make sense for organisms. For example, eagles and ostriches both have wings, but only the former use them for flying. As a result, we cannot say that “birds have wings in order to fly”. We could say that eagles have wings in order to fly, but this is not the case for ostriches. The reason for this is that the various types of wings were not intentionally designed for flying, but are the outcomes of natural evolutionary processes.

It is nevertheless important to note that teleology in general is not illegitimate in biology. Explanations based on natural selection exhibit a robust form of teleology, and it is legitimate to state that something exists for a purpose because it was selected for fulfilling it [56]: this can be described as selective teleology [54]. Rather, what is illegitimate is design teleology, the idea that something exists for a purpose because it was intentionally designed to fulfill it. This idea entails that entities have the features that they need for the roles or functions they were intended to fulfill; this is the case for artifacts but not for organisms. Therefore, what is rejected in biology is not the idea of teleology in general, but the idea of intended uses that are the outcome design, which we describe as design teleology [57]. It is this idea of design teleology that is of interest to us in this study.

One can assume that the ideas of design and purpose could make students intuitively think that there exist “genes for” traits, in the sense that genes exist for an intended use or purpose. Previous research has shown how this can be possible. For instance, in a study it was found that undergraduates used purpose-based reasoning to explain properties that have particular consequences. Although they were generally inclined to think of physical properties as inherited and stable, they explained differently those properties that performed some function or were useful in a particular habitat [58]. In our study, we wanted to investigate whether students implicitly associate words related to genetics with words related to goal-directedness. If students made these associations, then this could be evidence that they intuitively think that there exist “genes for” traits. This is the basis for our Hypothesis 1 (H1) below.

### Genetic essentialism

Essentialism is the idea that entities have essences, i.e. underlying properties that are characteristic of them. There are several distinct kinds of essentialism [59]; the one that is of interest for this study is psychological essentialism; the intuition that organisms have fixed essences that cannot undergo any change [60]. Several researchers have shown that children believe that organisms are characterized by underlying, distinctive “essences” that make them what they are [61–65]. Artifacts can be said to have essences, which are related to their intended use and are thus fixed. A chair will be a chair even if we use it as a table and put our meal on it; an airplane will be an airplane even if it does not fly but is stored in a museum. However, this is not the case for organisms, because they can evolve and undergo significant changes in their developmental potential, which could be considered as their essence [66].

The impact of essentialism on people’s understanding of genetics has already been investigated. It has been suggested that genetic essentialism may lead people to view genetically influenced traits as immutable and determined, to view the relevant genes as being the fundamental cause of the respective character or condition, to view groups that share a genetic character as being homogeneous and discrete, and to perceive characters or conditions as more natural if they are genetically determined [67,68]. The impact of essentialism on people’s perceptions of social concepts has been extensively studied and investigated in social sciences and social psychology. For example, several studies have shown that essentialism is used to explain category differences for race, gender, aggressiveness, criminality, sexual orientation, obesity, mental disorder [69–74], while some studies emphasize how essentialism takes its ground in genetics [67,75,76]. Another study investigated 40 different social categories, such as intelligence, race or religion, and suggested a bidimensional structure of essentialism consisting of entitativity and naturalness [77]. However, not many studies on psychological essentialism have focused on scientific concepts [53] and we thus aimed at contributing to this body of research.

Genetic essentialism has been conceptualised as comprising four dimensions: the homogeneity of genes within species, which downplays the variation among the members of the same species; the fixity of genes, which entails that they are transmitted immutable across generations; genes as internal, single causes, which means that they directly cause observable traits; and the inference for the presence of gene from a given physical or mental characteristic, which entails that the presence of a particular gene can be inferred from the observation of a related characteristic [67]. In our study, we have only investigated one of these dimensions, genes as immutable entities that we generally describe as “stability”. The reason for this is that it could be the case that students think that genes are stable, or fixed, considering that previous studies in biology education have shown that they have a poor understanding of the nature of mutations [78,79], even after instruction on mutations [80]. In particular, we investigated whether students associated genetics concepts with concepts related to stability, which is the basis for our Hypothesis 2 (H2) presented below.

#### Relation between teleology and essentialism

Previous research has concluded that essentialist and teleological conceptions about inheritance are contrasting: traits with some function are either more heritable and less modifiable (essentialist stance) or less heritable and more modifiable (purpose-based stance) [58]. Other studies, have provided evidence that students’ teleology and essentialism conceptions in the context of biology are not related [27,81]. Given these findings, in our study we assumed that students’ associations between genetics and goal-directedness concepts should not be correlated with their associations between genetics and stability concepts. This is the basis for our Hypothesis 3 presented below.

#### The impact of learner characteristics

Previous research has shown that the presence and “strength” of intuitions across several areas of science may also depend on a series of learner characteristics [82]. First, it is well-known that prior knowledge in a domain is a strong predictor of learning in general (*d* = 0.67), and of science learning in particular (*d* = 0.8, [83]). For a recent study highlighting the impact of prior knowledge on students’ achievement in biology (and chemistry), see [84]. Moreover, knowledge acquired from non-formal sources accounts for the origin of many scientific misconceptions and intuitions [85]. Second, gender has been reported to influence scientific achievement, e.g. studies showing a somewhat lower achievement of women in concept tests in physics (see, e.g., [86,87]; or to impact socio-scientific explanations, as women showed higher endorsement of genetic explanations for personality, intelligence, and success in life than men [88]). Finally, another recent study has found that the variable “age” influenced students’ belief in genetic determinism independently of and even more than the variable “knowledge of genetics” [89]. Henceforth, we investigated whether learner characteristics such as gender, age or previous learning of biology could impact secondary school students’ implicit association of genetics and teleology concepts, or students’ implicit association of genetics and essentialism concepts.

#### Research hypotheses

Based on the above research and considerations, in our study we aimed at testing the following hypotheses:

- Hypothesis 1 (H1): If design teleology leads to the preconception that some traits of individuals exist in order to perform an intended role, then in the context of genetics it might lead to the preconception that genetically influenced traits of individuals exist in order to perform an intended role and that they are determined by some inherent factors that exist for this purpose (*genes “for” traits*). In other words, we expect upper secondary students to implicitly associate genetics and teleology concepts. This hypothesis would be confirmed if in the implicit association test we found associations between genetics concepts and teleology (goal) concepts.
- Hypothesis 2 (H2): If psychological essentialism leads to the preconception that some traits of individuals are fixed and unchangeable due to some inherent factors, then in the context of genetics it might lead to the preconception that genetically influenced traits of individuals are fixed due to some inherent factors (*genes as essences*), making developmental processes to seem to have equally fixed outcomes. In other words, we expect upper secondary students to implicitly associate genetics and essentialism concepts. This hypothesis would be confirmed if in the implicit association test we found associations between genetics concepts and essentialism (stability) concepts.
- Hypothesis 3 (H3): Essentialist and teleological intuitions are independent, and so we expect to find no correlation between the intuitions according to H1 and H2.

## Methods

The Department of Public Instruction (“Département de l’Instruction Publique”) is the authority in charge of education in Geneva, Switzerland. The “Service of Research in Education” of the Department of Public Instruction is the institutional review board that considers the legal and ethical aspects of research investigations at schools in Geneva and approved our study (decision of October 7, 2015).

### Participants

Our study included 169 students, 15 to 18 years old (M = 16.2, SD = 0.80), from eleven different schools. Another 25 participants were eventually excluded, as either 10% or more of their answers fell below the cut-off of 300ms recommended by IAT studies, or they had more than 30% errors overall [90]. The students who participated in our study attended two different types of upper secondary schools in Geneva, Switzerland: “Formation gymnasiale” (denoted as ‘FG’) and “Formation de culture générale” (denoted as ‘FC’); the latter offers students a specialized professional training in either arts, communication, health or social education; while the former prepares students for formal university studies. According to the International Standard Classification of Education of the UNESCO, they both belong to ISCED level 34 called “upper secondary general education” [91,92]. The sample also included 45 French students coming from upper-secondary school named “Lycée” (denoted as ‘L’), that prepares students for formal university studies -similarly to ‘FG’- and which also belongs to ISCED level 34. The participants were 109 females and 60 males, with a majority of 105 students coming from “Formation gymnasiale”. Detailed characteristics of the sample are presented in Table 1.

**Table 1:** Summary of the sample’s characteristics. Entries are number of students.

Gender		School type			Age			
Male	Female	FG	FC	L	15	16	17	18
60	109	105	28	36	38	51	80	-

FG: “Formation gymnasiale”; FC: “Formation de culture générale”, L: “Lycée”.

### Apparatus

The IAT was administered on computers, through an online version developed by Harvard Project implicit (https://implicit.harvard.edu/). Project Implicit developed the implicit association test based on our design, provided us with raw data, and supported us for the whole duration of the project. Our study was conducted in French. The test required on average 15 minutes to be completed and no major administration issues occurred. The complete dataset is available in S4 Appendix, in accord with the open data requirements of the journal.

### Design

**Fig 1:**
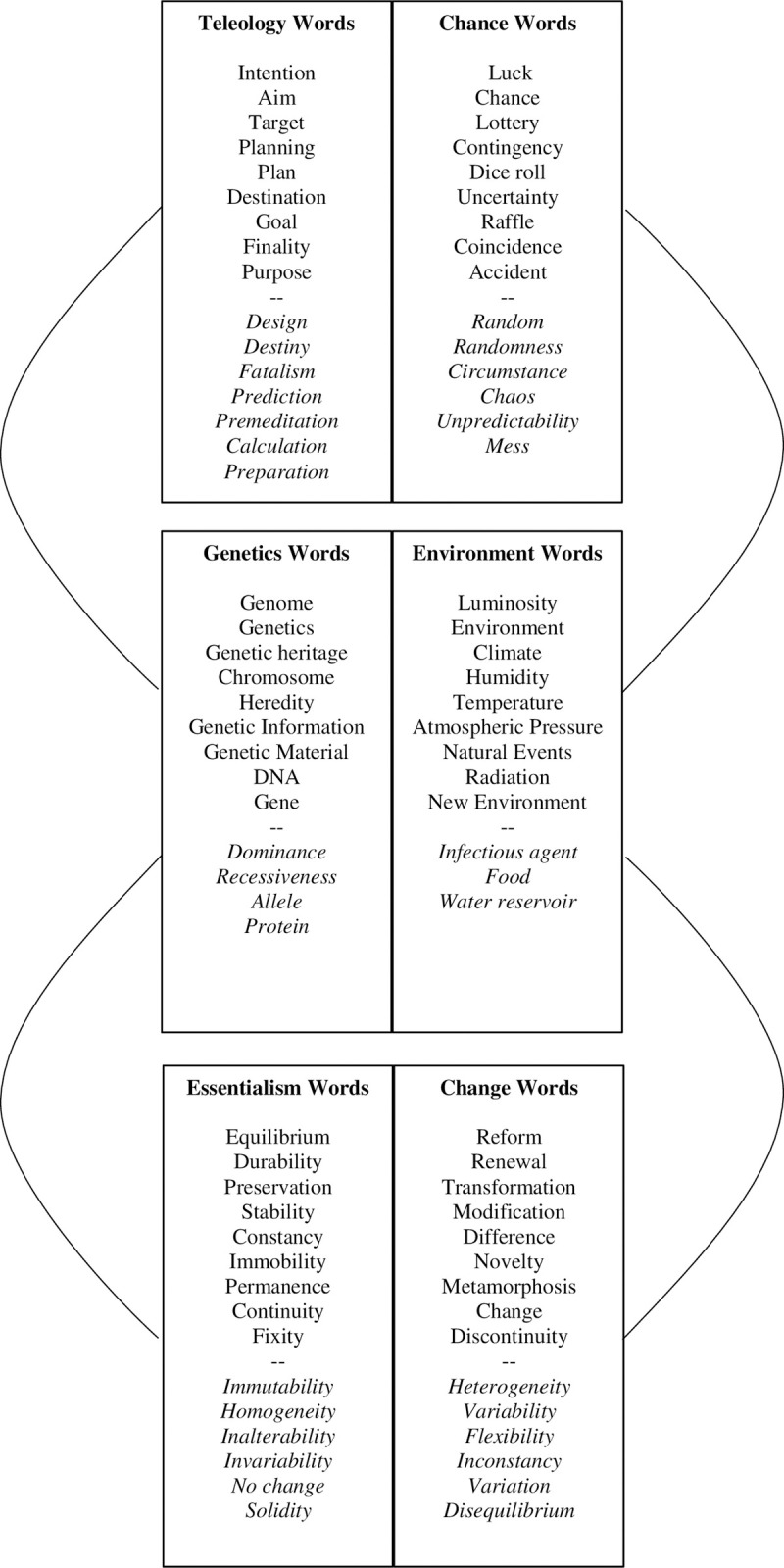
List of words used in the IAT. Words in italics were tested but not kept in the final version. The words presented here are our translation of the original list of French words that can be found in Supplementary Materials.

By definition, the IAT requires two pairs of contrasted categories (see above). In the present study, the IAT consisted of two parts: the first one being about teleology and genetics concepts (*Genetics & Teleology IAT*), and the second one being about essentialism and genetics concepts (*Genetics & Essentialism IAT)*. In the *Genetics & Teleology IAT*, the choice of the two pairs of contrasted categories were ‘Genetics *vs*. Environment’ and ‘Teleology *vs*. Chance’, because such a choice allowed to investigate the association between genetics and teleology concepts. In the *Genetics & Essentialism IAT*, the choice of the two pairs of contrasted categories were ‘Genetics *vs*. Environment’ and ‘Essentialism *vs*. Change’, because such a choice allowed to investigate the association between genetics and essentialism concepts. Because of the focus on genetics concepts, it should be noted that the contrast ‘Genetics *vs*. Environment’ is present both in the *Genetics & Teleology IAT* and in the *Genetics & Essentialism IAT*. Therefore this “shared” contrast could be seen as a baseline against which we computed the target associations. For each category, the terms were selected based on pilot testing and are shown in Fig 1 (the original list of French words can be found in Supplementary Materials—see S1 Appendix). More information about the pilot testing phase and the choice of categories can be found in Supplementary Materials (see S2 Appendix).

The IAT consists of several blocks of trials; from the different existing designs, we chose to follow the classical one with five blocks [51], that is described in more details in the procedure section.

### Procedure of constructing the IAT

To avoid any order effect, half of the students started with the first part, whereas the other half started with the second one. As already mentioned, the IAT measures the differential association of two target concepts (concepts related to genetics vs. concepts related to environment) with an attribute that appears in a second task (words indicating goal vs. words indicating chance in the case of teleology, and words indicating stability vs. words indicating change in the case of essentialism).

The rationale for the choice of words for the target concepts was the following: when it comes to genetics, a common misconception is that the influence of genes and of the environment can be clearly distinguished. However, this is not possible to do at the individual level and we can only statistically study differences in variation at the population level due to differences in genes or the environment. We therefore created a contrast between genes and environment for both the *Genetics & Teleology IAT* and the *Genetics & Essentialism IAT* in order to assess how much participants attribute to genetic or environmental influences.

For the attribute of the *Genetics & Teleology IAT* we created the contrast between goal and chance. Our hypothesis was that students would associate genetics concept with goals (teleology) rather than with chance (H1). Similarly, for the *Genetics & Essentialism IAT* we created the contrast between stability (one of the four dimensions of essentialism we decided to study) and change. Our hypothesis was that students would associate genetics concept with stability (fixity dimension of essentialism) rather than with change (H2). Eventually, the words included in Fig 1 are just translations of the French ones we used, which can be found in the Supplementary Materials (see S1 Appendix).

#### Genetics & Teleology IAT

In this first part, the aim was to explore associations between genetics and teleology concepts. As shown in Table 2, students were first asked in block 1 to classify genetics words (such as “DNA”, “gene”, “heredity”) *vs*. environment words (such as “natural events”, “climate”, “temperature”) by choosing the appropriate keys on the keyboard. After this, they were asked in block 2 to attribute teleology words (such as “goal”, “plan”, “target”) *vs*. chance words (such as “luck”, “dice roll” “accident”) to their respective category. In block 3, they were asked to use the same key to classify “genetics” and “goal” words, as well as another key for classifying both “environment” and “chance” words. In block 4, students were again asked to classify teleology vs. chance words. Finally, in block 5 students had to classify words where categories had switched with respect to block 3, by using the same key for “genetics” and “chance” words, and another key for both “environment” and “teleology”. Note that the order of blocks 3 and 5 were randomized, so that half of the students were given blocks in the following order: 1, 2, 3, 4, 5; while the other half were given blocks in the following order:1, 2, 5, 4, 3.

**Table 2:** Summary of the IAT blocks of trials for the ‘Genetics & Teleology’ association.

Block	Trials	Function	Items assigned to left-key response	Items assigned to right-key response
1	20	Practice	Teleology	Chance
2	20	Practice	Genetics	Environment
3	40	Test	Genetics & Teleology	Environment & Chance
4	20	Practice	Chance	Teleology
5	40	Test	Genetics & Chance	Environment & Teleology

#### Genetics & Essentialism IAT

In this second part, we aimed to explore associations between genetics and essentialism concepts. Similarly to the ‘Genetics & Teleology’ part, students were asked to classify words about ‘*genetics’* (such as “DNA”, “gene”, “heredity”), ‘*environment’* (such as “natural events”, “climate”, “temperature”), *‘essentialism’* (such as fixity, constancy, preservation) and ‘*change’* (such as transformation, modification, renewal), and the order of blocks 3 and 5 were randomized. The procedure is shown in Table 3.

**Table 3:** Summary of the IAT blocks of trials for the ‘Genetics & Essentialism’ association.

Block	Trials	Function	Items assigned to left-key response	Items assigned to right-key response
1	20	Practice	Essentialism	Change
2	20	Practice	Genetics	Environment
3	40	Test	Genetics & Essentialism	Environment & Change
4	20	Practice	Change	Essentialism
5	40	Test	Genetics & Change	Environment & Essentialism

Based on students’ performance during this part of the test, a D-score for each student was calculated. In the ‘Genetics & Teleology’ study, Compatible Response Latencies (CRL) were calculated from block 3 where ‘*genetics*’ and ‘teleology’ are paired according to our hypothesis; Incompatible Response Latencies (IRL) were computed from block 5 where ‘*genetics’* and ‘*chance*’ are paired. Note that the words “compatible” and “incompatible” refer to compatibility and incompatibility with the intuition to be investigated (i.e. that genetics is associated with teleology or essentialism). D-scores greater than 0 were interpreted as evidence of an association between genetics and teleology concepts (or chance and environment concepts). Similarly, in the ‘Genetics & Essentialism’ study, Compatible Response Latencies (CRL) are calculated from block 3 where ‘*genetics*’ and ‘*essentialism*’ are paired according to our hypothesis; Incompatible Response Latencies (IRL) are computed from block 5 where ‘*genetics’* and ‘*change*’ are paired. D-scores greater than 0 were interpreted as evidence of an association between ‘*genetics’* and ‘*essentialism’* concepts (or environment and change concepts).

### Learner characteristics (control variables)

As explained in the research background section, the following control variables were collected: “gender”, “age”, “class”, “previous learning of biology in school”, “previous learning of biology outside school”. The variable “previous learning of biology in school” was assessed by asking students whether they had formal teaching of biology at lower secondary school (12–15 years old) and/or upper secondary school (15–18 years old), according to ISCED standards [92]. Similarly, “previous learning outside school” was assessed by asking students whether they had informal knowledge of biology, such as from TV documentaries, movies, or the internet. All learner characteristics were measured before the test completion in a background information section.

### Statistical methods

First, a one-sample t-test was carried out in order to assess whether the obtained D-scores were significantly greater than 0 in order to answer H1 and H2. Second, an analysis of covariance [93] was made in order to investigate the influence of gender, age, class, and previous learning of biology in/outside school on D-scores. All statistical analyses were performed with the software R [94,95].

Due to hierarchical structure of our data (students are nested in class groups) one has to consider a multilevel model [93]. Note that considering multilevel modelling here is necessary because *not* doing so could lead to (strongly) distorted significance tests (*p* too small) and effects size values (*d* too large; note that the IAT D value is analogous to an effect size) [96,97]. In order to be sure that these distortions do not occur, or otherwise whether a hierarchical model is necessary or not, one needs to calculate the intra-class correlation (ICC), which represents the proportion of the total variability of the variable of interest (here: D-scores) that is attributable to the classes [93]. If the ICC is high, a large proportion of variance is attributable to the second (or higher) level of the hierarchical structure, and a multilevel analysis is the appropriate method.

Besides the ‘Genetics & Teleology’ and the ‘Genetics & Essentialism’ associations considered separately, we were interested in whether students’ teleological and the essentialist intuitions in the context of genetics were correlated, in order to answer H3. Correlations between D-scores from both IAT tests were calculated as Pearson correlation coefficient and tested for significance with a Pearson correlation test. Note that a correlation between D-scores is a second order association in the sense that D-scores themselves are already a measure of association.

## Results

In this section, we report the analysis of the ‘*Genetics & Teleology IAT’* and *‘Genetics & Essentialism IAT’*. An implicit association is characterized by D-scores; the higher is a student’s D-score, the stronger he or she exhibits the association between genetics and teleology concepts. An average D-score significantly larger than zero indicates a tendency of the target age group of secondary students for the association in question. Additionally, we provide the distribution of D scores for the to IATs in the Supplementary Materials (see S3 Appendix), as the more complete distributional information could be of interest for future research [97,100].

### Hierarchical data structure

The test for the necessity of multilevel analysis with students as level 1 and classes as level 2 yielded an ICC not significantly different from 0 for the ‘Genetics & Teleology’ association and of 0.04 for the ‘Genetics & Essentialism’. Henceforth, it is justified to use methods of analysis without taking into account the hierarchical data structure.

#### Genetics & Teleology IAT

More than half of the students (58%) were faster in associating genetics and teleology (goal) or environment and chance, rather than the opposite (genetics and chance or environment and teleology). The average D-score in the sample was slightly but significantly greater than 0 (M = 0.07, SD = 0.34; *t*(168) = 2.56, *p*<0.001). We thus found upper secondary students to have a slight association of genetics and teleology concepts, in agreement with hypothesis H1.

#### Genetics & Essentialism IAT

About two out of three students (67%) were faster in associating genetics and essentialism (stability) or environment and change, rather than the opposite (genetics and change or environment and essentialism. The average D-score in the sample was slightly but significantly greater than 0 (M = 0.14, SD = 0.37; *t*(168) = 4.83, *p*<0.001). We thus found upper secondary students to have a slight association of genetics and essentialism concepts, in agreement with hypothesis H2.

### Correlation of Genetics & Teleology and Genetics & Essentialism associations

In this section, we report the analysis of a potential correlation between students’ *Genetics & Teleology* D-scores and students’ *Genetics & Essentialism* D-scores. We expect secondary school students not to exhibit such an association according to Hypothesis H3. *Genetics & Teleology* D-scores and *Genetics & Essentialism* D-scores were not correlated, according to a non-significant Pearson correlation test (*r*(169) = 0.11, *p* = 0.14). For more details, the scatterplot of the ‘*Genetics & Teleology’* D-scores vs ‘*Genetics & Essentialism’* D-scores can be found in Supplementary Materials (see S3 Appendix). In other words, the tendency to associate Genetics and Teleology concepts is not associated with the tendency to associate Genetics and Essentialism concepts.

### Impact of learner characteristics

In this section, we report the analysis of the impact of learner characteristics on the tendency to associate genetics and teleology concepts, or on the tendency to associate genetics and essentialism concepts. Such characteristics may have, or may not have an impact. For the ‘Genetics & Teleology IAT’ and the ‘Genetics & Essentialism IAT’ studies, the analysis of covariance supported no significant effect of gender, age, school class, previous learning in school nor previous learning outside school (*F*(20,148) = 0.93, *p* = 0.54, and *F*(20,148) = 1.51, *p* = 0.08 respectively).

## Discussion

### Main findings

In this study we developed an IAT in order to test the following hypotheses: that secondary students would implicitly associate genetics and teleology (goal-directedness) concepts (H1); that they would also implicitly associate genetics and essentialism concepts (H2); and that there would be no correlation between these two associations (H3). We also investigated the potential effect of particular control variables on these associations.

First, our results provide evidence for an implicit association between genetics and teleology (goal) concepts rather than between genetics and chance concepts, in agreement with H1. While this association is of moderate strength, it is something that is worth further investigating in the future: if students associate genetics concepts with teleological (goal-directedness) concepts, they might be thinking that genes exist for a goal, which has implications for teaching and learning genetics. Genes that perform functions may exist, but these are not the outcome of purposeful design.

Second, we have found an implicit association between genetics and essentialism (stability) concepts rather than between genetics and change concepts, in agreement with H2. This is again an association of moderate strength, but also worth investigating further in the future. If students associate genetics concepts with stability concepts, then they might be thinking that genes cannot change, which again has implications for teaching and learning genetics. In fact, genes do change because of mutations and this is how new variations come to exist, as basis for evolution.

Our findings regarding H1 are in agreement with previous research findings such as those by Gould and Heine [53], who found that *people implicitly associated genes with fate more than they did with choice*. In our study we found that students were overall found to associate genetics concepts both with teleology (goal-directedness) and with essentialism (stability) concepts. These findings on their own do not answer the question of how teleology and essentialism might be obstacles for understanding genetics, but we think that they justify further investigation of this question. Previous research has shown that students can exhibit both essentialist and teleological reasoning related to inheritance, as they may use purpose-based reasoning to explain properties performed some function or were useful in a particular habitat but also generally consider physical properties as inherited and stable [58].

This same study also concluded that essentialist and teleological conceptions about inheritance are contrasting: traits with some function are either more heritable and less modifiable (essentialist stance) or less heritable and more modifiable (purpose-based stance). It is therefore reasonable to anticipate no correlation between the implicit association of teleology and genetics concepts and that between essentialism and genetics concepts, something also found in previous research [27,81]. Indeed this is what we found, confirming our Hypothesis 3. It should be noted, however, that it is also limited by the conceptual focus we have chosen in this study. It could be possible that with a specific social focus (e.g., when making sense of gender or races differences), teleological explanations using genetics terms would be associated with essentialist explanations using genetic terms. Further research would be required to understand better how strongly teleology and essentialism explanations depend on a specific research focus.

Additionally, the analysis of learners’ characteristics supports that none of these (gender, age, school class, previous learning of biology in school, previous learning of biology outside school) seems to have an influence on associating genetics and teleology concepts, or on associating genetics and essentialism concepts. To the best of our knowledge, there is no other past study that investigated the influence of such variables on these associations. One reason for the lack of influence of the learners’ characteristics would be that teleological and essentialist thinking, here with a focus on genetics, may be deeply-rooted among humans with diverse characteristics (gender, age, previous learning in/outside school). We are aware that such a generalization would require a cross-cultural study using a larger random sample. However, it is also corroborated by other research providing evidence that, beyond the genetics context, the teleological and essentialist intuitions are quite universal: on the one hand teleological thinking is present at all ages, among children, teenagers and students [23,98] and even among physical scientists [5], and also among non-western populations, such as Chinese people [99]. On the other hand, essentialism also seems to be present at all ages [60], and has been observed among populations from various cultural background such as Mongolian adults, Indian adults or Mexican children [100].

### Limitations of the study

Some limitations of the present study have to be noted. First, administering a single-target IAT [101] over an additional sample could have provided some additional information and confirmation of the strength of the associations between genetics and teleology concepts, as well as the association between genetics and essentialism concepts. Second, regarding the ‘Genetics & Essentialism’ association, we focused only one dimension of essentialism (stability) in order to explore the relevance of the IAT in that context. Other dimensions of genetic essentialism such as the homogeneity of genes within populations, the causal aspect of genes, and the inference of genes from traits [67] could be studied to arrive at a more fine-grained analysis of the links between genetics and essentialism concepts.

We should also note that we are aware of the more general criticisms of the IAT. First, it has been argued that the IAT has low validity [102,103]. However, the internal, construct, and predictive validity of the inferences based on the implicit association test have been reported by several studies [46,47]. In particular, a meta-analysis of 122 research reports supports the predictive validity of the IAT [45]. Second, there exist several factors likely to bias the measurement of the association, such as individual cognitive skills, the familiarity and perceptual similarity of stimuli [104], or the context of the study [103]. Finally, it has also been suggested that it is possible to fake the IAT on purpose, and that experts are unaware of the faked datasets [105]. However, the second and third criticism are not specific to the IAT (or other implicit methods), but they apply as well e.g. to questionnaires and interviews. Moreover, the IAT has undergone thorough testing and development over many years now, leading to a set of “best practices” that answer many of the criticisms initially raised [30].

### Implications for teaching

In sum, our study has provided further evidence for the human propensity to think in teleological and essentialist terms. We recommend that biology teachers should be taught about that, and that they should address the respective erroneous intuitions in their teaching. This means that simply teaching genetics concepts is not enough. Teachers should rather also address teleological and essentialist intuitions, both generally and specifically in the context of genetics.

In case students think that genes exist for a purpose, teachers could explain that it is a selection teleology and not a design teleology that is legitimate in this case. This means that genetics education should emphasize that genes that perform a function were selected (along with their bearers) because of the positive consequences of this function but that those genes were not intentionally designed or co-opted. Teachers should make clear that genes do not exist for a purpose, as many genes contribute to the same phenotypic outcomes and the relation between genes and traits is a many-to-many one.

In case students think that genes are immutable, teachers should explain that they can change and that the changes they might undergo are unpredictable. The fact that both heredity and development are processes with robust outcomes does not entail that genes cannot change.

Teachers should emphasize that neither genes, nor the traits in the development of which they are involved do not necessarily remain fixed. Genes themselves may change; their expression may also change depending on the environment.

Future research should look into whether (and how) teaching interventions that address students’ genetic teleology and genetic essentialism conceptions have any impact in their understanding of heredity and of the role of genes. Furthermore, the GET-IAT presented here could be used together with a genetics concept inventory to explore if there is a correlation between the two. In such a case, the GET-IAT could serve to predict the actual understanding or knowledge of genetics and the related performance in the classroom.

## Supporting information

S1 Appendix List of IAT words used in original language (French). (DOCX)

S2 Appendix Choice of categories and terms & Pilot studies. a. Choice of Categories and Terms for the Present Study. b. Pilot Studies, *Pilot Study 1 (P*_*1*_*)*, *Pilot Study 2 (P*_*2*_*)*, c. Note about the sample of the main study.
(DOCX)

S3 Appendix D-score distributions and scatterplot. (DOCX)

S4 Appendix (CSV)
