# Supplementary material for: Implicit associations of teleology and essentialism concepts with genetics concepts among secondary school students
Source: PLoS One. 2020 Nov 20;15(11):e0242189. doi: 10.1371/journal.pone.0242189 (PMC7679004; doi:10.1371/journal.pone.0242189)
Supplement: S1 Appendix — (DOCX) [file pone.0242189.s001.docx]

**S1 Appendix: List of IAT words used in original language (French).**
