# Supplementary material for: Implicit associations of teleology and essentialism concepts with genetics concepts among secondary school students
Source: PLoS One. 2020 Nov 20;15(11):e0242189. doi: 10.1371/journal.pone.0242189 (PMC7679004; doi:10.1371/journal.pone.0242189)
Supplement: S2 Appendix — a. Choice of Categories and Terms for the Present Study. b. Pilot Studies, Pilot Study 1 (P1), Pilot Study 2 (P2), c. Note about the sample of the main study. (DOCX) [file pone.0242189.s002.docx]

**S2 Appendix: Choice of Categories and Terms & Pilot Studies**

**a. Choice of Categories and Terms for the Present Study**

In past IAT studies, there was usually a clear distinction between targets (which are nouns) and attributes (which are adjectives). In our study both the targets and the attributes were nouns. The targets were genetics concepts, whereas the attributes were teleology and essentialism concepts. Therefore, as our approach is conceptual, we use the more generic term “category” to refer either to target or attribute in the rest of the article.

First, for each category of concepts, a contrast (i.e. two opposite concepts, such as ‘*good*’ vs ‘*bad*’) is required due to the IAT design. Even though it has been shown the contributions of both nature (anything related to our genetic constitution) and nurture (anything related to our development and upbringing) cannot actually be scientifically disentangled on the conceptual level, it is possible to investigate whether students tend to associate nature (here, genetics) or nurture (here, environment) with teleology or essentialism concepts. In their study, Gould and Heine (2012) chose to contrast genetics and *socialization*, whereas we chose to contrast genetics and *environment*. Socialization mostly relates to behaviors (such as experience, training, nurture), whereas environment is mostly about the natural conditions that might affect life (such as humidity, climate, temperature). Second, our choice of the teleology category, though slightly different, is closely related to Gould and Heine’s choice of *fate*. However, these authors chose to contrast fate and *free-will*, whereas we chose to contrast teleology with *chance*. Again, free-will characterizes human behavior, whereas chance also relates to unconscious processes. Third, the last pair of contrasted categories we considered in our study is about essentialism and *change*, which was not investigated by Gould and Heine. Genetic essentialism was characterized in another study in different dimensions; among these we focused on the idea that “genes are fixed”, which is a misconception as random mutations affect all living organisms, resulting in changes in genes. However, biology education research has revealed a poor understanding of random mutation and more generally of randomness; for this reason, we wanted to investigate further whether the “stability of genes” misconception could also be found out using implicit tools, such as the IAT.

In sum, Gould and Heine seem to take a behavior-centered perspective in their choices of categories; in contrast, we adopted a conceptual-based approach. Therefore, the names of our categories are: genetics vs environment, teleology (in the sense of goal) vs chance, and essentialism (in the sense of stability) vs change. It must be noted that students were usually not familiar with the terms teleology and essentialism. In order to facilitate students’ understanding, *during the IAT tasks in block 1, 2, 3 ,4, 5 only*, the terms ‘teleology’ and ‘essentialism’ were therefore respectively replaced by the more understandable terms ‘goal’ and ‘stability’. However, throughout this article, we refer to ‘teleology’ and ‘essentialism’, rather than ‘goal’ and ‘stability’.

While Gould and Heine used the IAT to investigate implicit associations between genetics and fate concepts, another study analysed the propensity of teleological and essentialist thinking in biology with explicit measures. In our study, we combined both approaches by using a different version of the Implicit Association Test used in the first study, and the specific kind of intuitive associations investigated in the second one.

**b. Pilot Studies**

*Pilot Study 1 (P_1_)*

The IAT was administered to 216 students in P_1_, and was followed by short group interviews with students. The aim of pilot study P_1_ was to develop an understandable list of words for students, suitable to be used in the IAT. That means, we had to develop words whose meaning are known by students and that make sense to be classified in a given category. For example, DNA is known by students, and it makes sense to classify DNA in the “genetics” category. Words were chosen for each of the following the categories: “genetics”, “environment”, “teleology”, “chance”, “essentialism” and “change”. Thus, in the first class we visited, we administered the IAT with a certain list of words (“L_1_”). After administration, we realized that some words in L_1_ were too complicated for students, therefore we replaced these too complicated words by some easier words, resulting in a new list of words (“L_2_”). Then, in the second class we administered the IAT with this new and “improved” list of words L_2_. This process eventually converged to a list of words that was understandable by a vast majority of students, that can be found in Fig 1.

Through this process, we concluded that the test was generally understood; however some words were considered to be too technical, such as the words “fatalism”, “dominance”, “immutability”, and where therefore replaced by other words. It should also be noted that “dessein”, the French word for “design” that is of great importance in our study, is less commonly used in French and its meaning was often unknown by students. Additionally, the final list of words (obtained after convergence towards an understandable list of words) was submitted to 3 senior biology education researchers who expressed their opinion about the relevance of the words appearing in the different categories: 87% of the words were deemed to fit their own categories by all three experts. Based on all this, we revised the list of words, as shown in Fig 1.

*Pilot Study 2 (P_2_)*

The aim of Pilot study P_2_ was to confirm on another sample that the words eventually chosen at the end of Pilot study P_1_ were understandable. Therefore, the list of words that emerged from P_1_ was used in the test that was administered to another sample of 86 students in P_2_. These were given a paper version of the list of words and were asked to write whether they knew the meaning of each word or not, as well as if they thought that it belonged or not, to the respective assigned category. Words were understood by 90% percent of the students and 85% of the words were deemed as fitting the category we had assumed they belonged to. As no major difficulties of understanding and classification of the words was found in this phase, we decided to keep this list of words presented in Fig 1.

Besides, the pilot studies served to know how much time it took students complete the test, and check whether any unexpected problems occurred during the administration of the test.

**c. Note about the sample of the main study**

It should be noted that the term “change of environment” (belonging to the “environment” category) was used during the administration to the first 71 students. We then noticed that it could be confusing, as this word could be associated with the “change” category or the “environment” category. Therefore, we replaced it with the term “new environment” during the version of the test administered to the remaining 98 students taking part in the study. In order to be sure that this change of words does not impact students’ D-scores, we compared the D-scores of the first subsample (71 students, using the word “change of environment”) and the second subsample (98 students, using the word “new environment”). It did not provide significant differences; therefore, we decided to merge the first sample (71 students) and the second sample (98 students) in a single one (169 students), on which the results of the present articles are based.
