# Supplementary material for: Implicit associations of teleology and essentialism concepts with genetics concepts among secondary school students
Source: PLoS One. 2020 Nov 20;15(11):e0242189. doi: 10.1371/journal.pone.0242189 (PMC7679004; doi:10.1371/journal.pone.0242189)
Supplement: S3 Appendix — (DOCX) [file pone.0242189.s003.docx]

**S3 Appendix: D-score distributions and scatterplot**

Below, we present the D-score distributions of the two IAT tests (Fig. 2,3). Such distributional information is much more complete than means and standard deviations (e.g. giving hints about skewedness, heavy tails, outliers etc.). It might be useful for further research to add this more complete information as it is known to be helpful especially in new areas and applications (1), which is true for our study (few applications of the IAT to conceptual intuitions so far), and more generally to the IAT in general. In the same perspective, the scatterplot for 'Genetics & Teleology' D-scores and 'Genetics & Essentialism' D-scores is given in Fig. 4.

1. Tukey J. Exploratory Data Analysis. Addison‐Wesley Publishing Company Reading, Mass. — Menlo Park, Cal., London, Amsterdam, Don Mills, Ontario, Sydney 1977, XVI, 688 S. - Beyer - 1981 - Biometrical Journal - Wiley Online Library


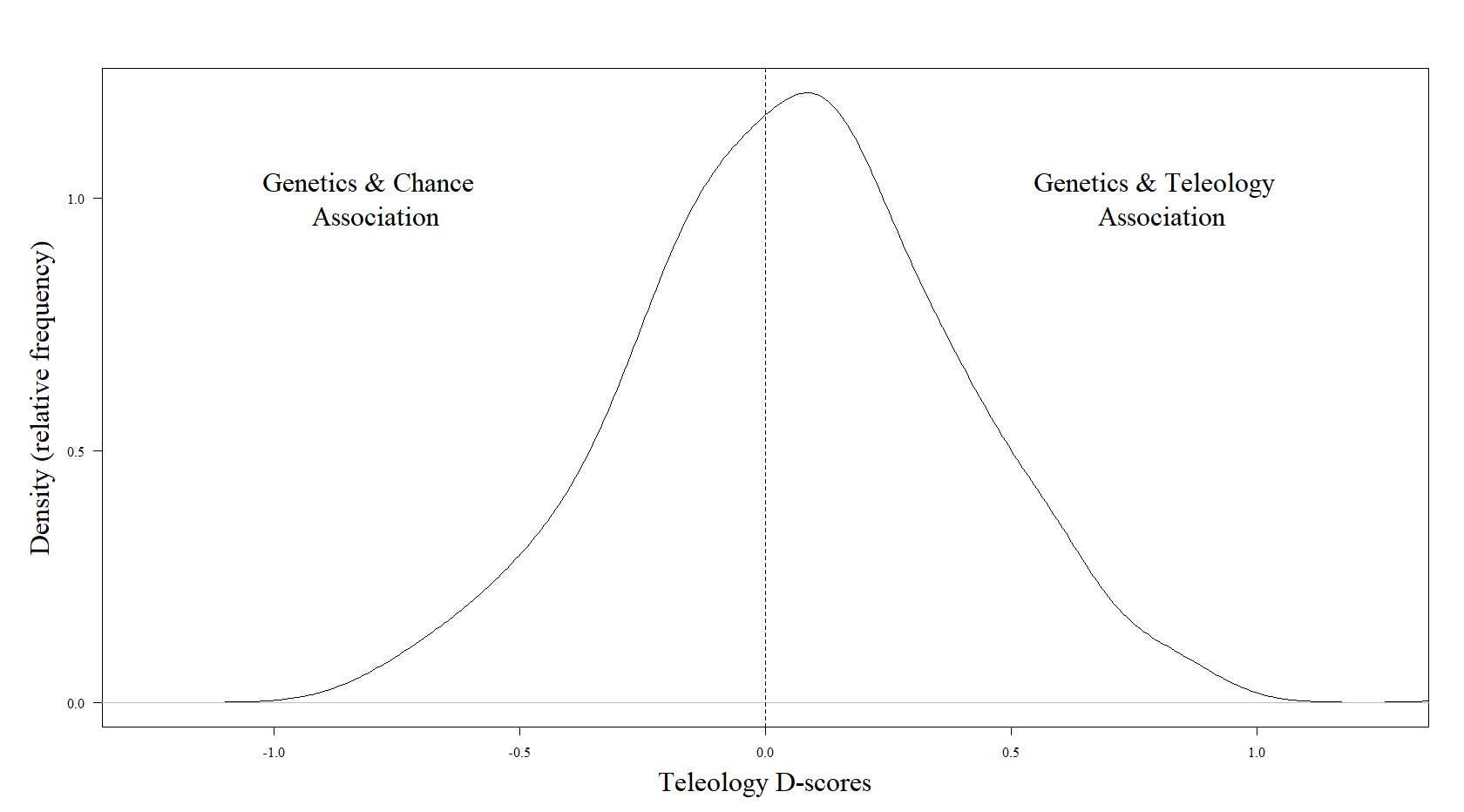


Fig. 2 Students’ distribution of D-scores. Positive D-scores show association of genetics and teleology (or, environment and chance); negative D-scores show association of genetics and chance (or, environment and teleology).

The right shift of the curve in Fig. 2 indicates that there are more positive D-scores than negative D-scores. As a positive D-score implies the presence of a “Genetics & Teleology” association, and a negative D-score implies the presence of a “Genetics & Chance” association, we conclude that students exhibit more often the “Genetics & Teleology” association than the “Genetics and Chance” association.


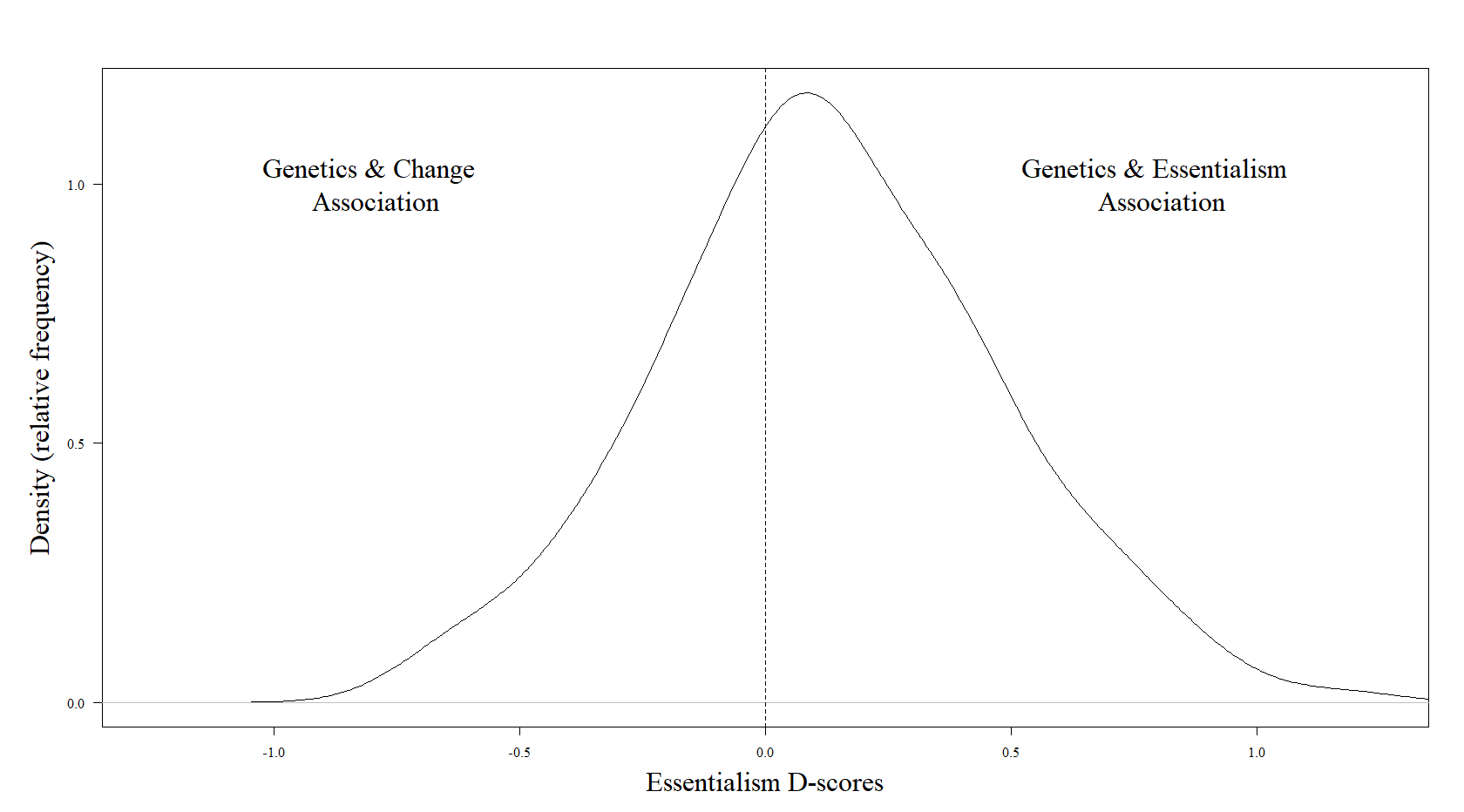


Fig. 3. Students’ distribution of D-scores. Positive D-scores show association of genetics and essentialism (or environment and change); negative D-scores show association of genetics and change (or, environment and essentialism).

The right shift of the curve in Fig. 3 indicates that there are more positive D-scores than negative D-scores. As a positive D-score implies the presence of a “Genetics & Essentialism” association, and a negative D-score implies the presence of a “Genetics & Change” association, we conclude that students exhibit more often the “Genetics & Essentialism” association than the “Genetics and Change” association.


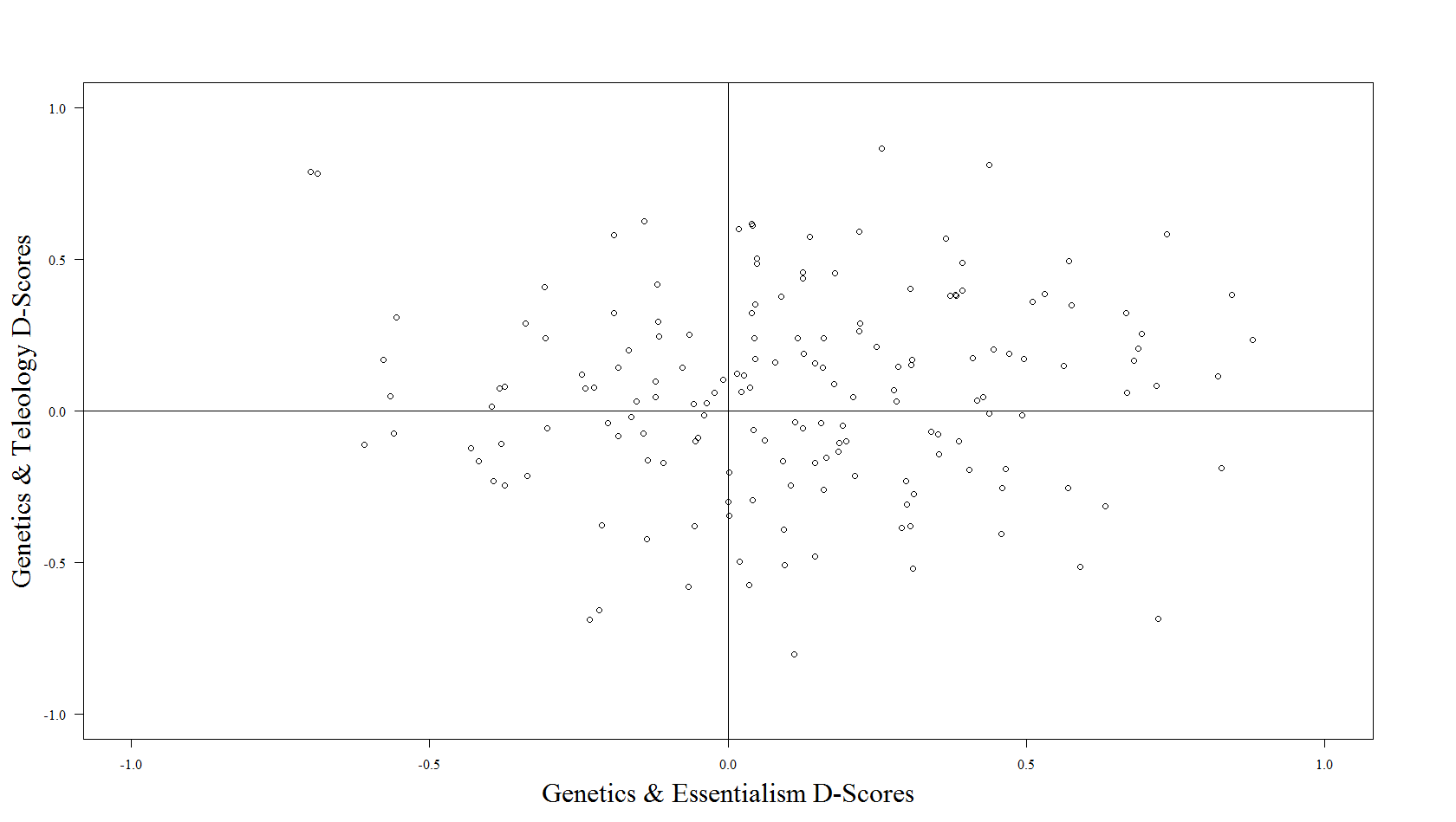


Fig. 4. Students’ joint distribution (scatterplot) of 'Genetics & Teleology' D-scores and 'Genetics & Essentialism' D-scores.

In Fig. 4, there are slightly more people on the top-right corner (D>0 for both parts), which is another way of looking at the data. However, while Fig. 2 and Fig. 3 only showed densities, Fig. 4 gives more detailed information about individuals with respect to their D-score in both parts. The absence of an obvious regular pattern supports the conclusion that there is no correlation between teleology D-scores and between essentialism D-scores.
